# Clinicopathological Profile and Real-World Survival Outcomes in Pure Uterine Sarcomas

**DOI:** 10.7759/cureus.104294

**Published:** 2026-02-26

**Authors:** Ritu Ahlawat, Divya Vuppu, Pranidhashree C. A, Anusree K, Priya Bhati

**Affiliations:** 1 Gynecological Oncology, Amrita Institute of Medical Sciences, Kochi, IND; 2 Biostatistics, Amrita Institute of Medical Sciences, Kochi, IND

**Keywords:** endometrial stromal sarcoma, leiomyosarcoma, recurrence pattern, survival outcome, uterine sarcoma

## Abstract

Background

Pure uterine sarcomas are rare and biologically aggressive malignancies with diverse histologic subtypes and heterogeneous clinical behavior. Owing to their low incidence, prospective data guiding optimal management and prognostic stratification remain limited, particularly in real-world settings. Evaluating survival outcomes and recurrence patterns in institutional cohorts is essential to better understand prognostic factors, treatment associations, and disease behavior. This study aimed to analyze survival outcomes, recurrence patterns, and clinicopathological factors influencing prognosis in patients with uterine sarcoma treated at a tertiary care center.

Methods

We conducted a retrospective analysis of 81 patients with histologically confirmed pure uterine sarcoma as treated during the 10-year period of 2011-2020. Carcinosarcoma was not included in this study. Clinical details, histologic subtype, stage, surgical and adjuvant treatments, recurrence, and survival outcomes were assessed. The Kaplan-Meier method was used to estimate overall survival (OS) and disease-free survival (DFS), and univariate analysis was performed to identify factors associated with prognosis.

Results

The mean age at diagnosis was 48 years, and most patients were multiparous and postmenopausal. The most common histologic type was leiomyosarcoma (48.1%), followed by low-grade endometrial stromal sarcoma (ESS) (29.6%). Stage I was the most frequent (55.6%). About 32.1% received chemotherapy, 9.9% radiotherapy, and 7.4% hormonal therapy; half received no adjuvant treatment. Recurrence was observed in 44.4% of cases, most commonly involving the pelvis and lymph nodes, with the lungs and liver also affected. Median OS and DFS were 72 and 70 months, respectively. Patients with low-grade ESS and early-stage disease had better survival. Chemotherapy was associated with higher recurrence. Histologic subtype, stage, and adjuvant treatment significantly influenced outcomes.

Conclusion

Histologic subtype, disease stage, and type of adjuvant therapy were independently associated with survival and recurrence outcomes in our cohort of uterine sarcoma patients. Early-stage disease and low-grade tumors demonstrated more favorable survival patterns. Given the rarity and heterogeneity of uterine sarcomas, real-world survival analyses such as this study help refine prognostic stratification and inform individualized treatment strategies. These findings provide clinically relevant insights that may guide risk-adapted management in this challenging group of malignancies.

## Introduction

Uterine sarcomas are rare but highly aggressive mesenchymal tumors, representing approximately 1-3% of all gynecologic cancers and 3-7% of uterine malignancies [1]. The incidence of uterine cancers, including both carcinomas and sarcomas, has been rising, likely driven by urbanization, lifestyle changes, and delayed age at first childbirth [2,3]. While endometrial adenocarcinoma is the most common histological type, uterine sarcomas represent a rare yet highly aggressive subset, accounting for approximately 3-7% of all uterine malignancies [4,5].

Pure uterine sarcomas encompass several histological subtypes, including leiomyosarcoma (LMS), endometrial stromal sarcoma (ESS; low- and high-grade), adenosarcoma, and undifferentiated uterine sarcoma (UUS). Carcinosarcoma, previously grouped with sarcomas, is now recognized as a metaplastic carcinoma and is managed according to endometrial carcinoma protocols [4-6]. LMS is the most common subtype (40-60%), followed by ESS (15-30%), adenosarcoma (5-15%), and the rare UUS [5,6].

Recurrence and survival vary widely by histology: LMS recurs in over half of cases, with a five-year overall survival (OS) of approximately 38%; low-grade ESS has favorable outcomes (>80% OS), while high-grade ESS and UUS are associated with poorer survival (38-54%). Adenosarcoma generally has an intermediate prognosis, which worsens in the presence of sarcomatous overgrowth. Across all subtypes, stage at diagnosis remains the strongest predictor of outcome [5-7].

Surgical resection, typically total hysterectomy with or without bilateral salpingo-oophorectomy (BSO), remains the mainstay of treatment. Adjuvant therapy is tailored according to histology: chemotherapy is primarily used for LMS and other high-grade tumors, radiotherapy may reduce local recurrence, and hormonal therapy is effective in low-grade ESS due to hormone receptor expression [5-7].

Despite these advances, uterine sarcomas remain rare and aggressive, with limited prospective data. Many prior studies included carcinosarcoma, which skews outcome interpretation. Accurate diagnosis is challenging, often requiring immunohistochemistry or molecular testing, and preoperative distinction from leiomyoma is frequently difficult [7,8]. Misclassification not only biases reported outcomes but can also lead to inadvertent morcellation, adversely affecting prognosis [9]. Although emerging techniques such as artificial intelligence (AI)-assisted diagnostics show promise, treatment strategies remain heterogeneous due to the rarity of these tumors [10]. Relapse is common across most subtypes except low-grade ESS, underscoring the need for evaluation of therapeutic approaches and real-world survival outcomes.

The present study evaluates the clinicopathological profile and real-world survival outcomes of patients with uterine sarcoma. By excluding carcinosarcoma, it provides a more precise and contemporary assessment of pure uterine sarcomas. Focusing exclusively on these tumors allows clearer characterization of their clinicopathological features and treatment outcomes, addressing a critical gap in the literature and generating real-world data to inform evidence-based management strategies.

## Materials and methods

This was a retrospective, single-center cohort study conducted in the Department of Gynecological Oncology at Amrita Institute of Medical Sciences, Kochi, Kerala, India, following approval from the institute's Institutional Ethics Committee (approval number: IEC-AIMS-2025-GYONC-246). The requirement for informed consent was waived due to the retrospective nature of the study.

All patients with histologically confirmed uterine sarcoma who underwent treatment at our center between January 2011 and December 2020 were included.

Patients referred from outside institutions after prior incomplete surgery (including myomectomy, subtotal hysterectomy, or partial tumor resection) were also included. For such patients, baseline clinicopathological details and operative records were reviewed, and survival time was calculated from the date of initial surgery performed at the outside institution.

Exclusion criteria included the following: absence of definitive histopathological confirmation, diagnosis of carcinosarcoma (as per WHO classification, categorized under uterine carcinomas), incomplete hospital records, and missing follow-up data.

Histological subtypes were defined according to the current WHO classification of uterine sarcomas [4-6].

Histopathological diagnosis was based on morphological evaluation supplemented by immunohistochemistry where indicated. LMS cases were confirmed using smooth muscle markers such as desmin, smooth muscle actin (SMA), and h-caldesmon. ESS demonstrated CD10 positivity, with low-grade ESS frequently expressing estrogen and progesterone receptors. High-grade ESS and UUS were diagnosed using appropriate immunomarker panels to exclude other mesenchymal or epithelial malignancies.

As per institutional protocol at Amrita Institute of Medical Sciences, histopathological slides from patients who underwent surgery at outside institutions were re-evaluated by a dedicated gynecologic oncopathologist to ensure uniform pathological assessment across the cohort. All cases were discussed in the institutional multidisciplinary tumor board at key decision points, including initial treatment planning, management following incomplete surgery, and the time of recurrence, to standardize therapeutic decisions.

Clinical and pathological data were retrieved from institutional electronic medical records. Where necessary, additional information was obtained from the pathology department and institutional cancer registry to minimize missing data. All data were anonymized and collected using a predefined data extraction template. Variables recorded included demographic and clinicopathological parameters such as age, parity, menopausal status, comorbidities, histological subtype, FIGO (International Federation of Gynecology and Obstetrics) stage, presence of residual disease, type of adjuvant therapy, recurrence characteristics, and survival outcomes.

Definitions

Residual Disease

Residual disease was defined as any macroscopic tumor remaining within 30 days after initial surgery. For patients referred after incomplete surgery elsewhere, residual disease status was determined based on operative findings at re-exploration and/or imaging (contrast-enhanced computed tomography (CECT) or positron emission tomography-computed tomography (PET-CT)). Absence of visible or histologically confirmed tumor at definitive surgery was classified as no residual disease.

Recurrence

Recurrence was defined as radiological or histopathological evidence of tumor reappearance after the documentation of complete remission on imaging (CECT or PET-CT). Sites of recurrence were categorized as pelvis (vaginal vault or pelvic soft tissue), lymph nodes (pelvic or para-aortic), abdomen (intra-abdominal organs excluding peritoneal surfaces and liver), peritoneum (abdominal and pelvic peritoneal surfaces), lung, and liver. Recurrences at multiple sites were recorded separately.

Survival Outcomes

Time-zero for survival analysis was defined as the date of primary surgery.

Overall survival (OS) was defined as the time from the date of primary surgery to death from any cause or last follow-up.

Disease-free survival (DFS) was defined as the time from the date of primary surgery to the first documented recurrence or death, whichever occurred first.

Patients who were alive without recurrence were censored at the date of last follow-up.

For patients referred after surgery performed at outside institutions, survival time was calculated from the documented date of their initial surgery.

Sample size

Based on an earlier study reporting a two-year OS of 63.3% in uterine sarcoma [11], with an absolute precision of 11% and a 95% confidence level, the minimum required sample size was estimated to be 74 patients.

Statistical analysis

Statistical analyses were performed using IBM SPSS Statistics for Windows, Version 26.0 (IBM Corp., Armonk, New York, United States). Categorical variables were summarized as frequencies and percentages and continuous variables as mean±standard deviation.

Associations between categorical variables were assessed using the chi-squared test.

Survival probabilities for OS and DFS were estimated using the Kaplan-Meier method, and comparisons between groups were performed using the log-rank test.

Variables demonstrating statistical significance (p<0.05) on univariate analysis were entered into multivariable Cox proportional hazards regression using a backward conditional method to construct a parsimonious model. Adjusted hazard ratios (HR) with 95% confidence intervals (CI) were reported. The proportional hazards assumption was assessed and satisfied.

A two-sided p-value of <0.05 was considered statistically significant.

## Results

Clinicopathological characteristics of the cohort

**Figure 1:**
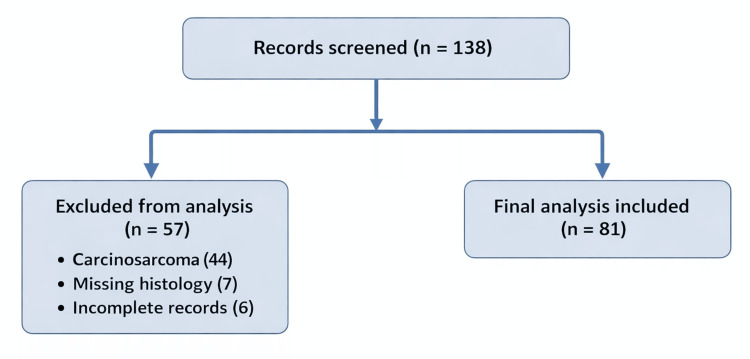
Flow diagram illustrating patient screening, exclusions, and final inclusion in the study cohort

A total of 81 patients with histologically confirmed uterine sarcoma treated between 2011 and 2020 were included in the final analysis after the exclusion of carcinosarcoma and cases with incomplete records (Figure 1).

The mean age was 48±12 years, and the mean BMI was 24±4.01 kg/m². Most patients were multiparous (n=64; 79%), and nearly half were postmenopausal (n=39; 48.1%). The most common presenting symptoms were abdominal pain (n=32; 39.5%) and abdominal mass (n=21; 25.9%), followed by abnormal uterine bleeding (n=16; 19.8%) and postmenopausal bleeding (n=12; 14.8%).

**Figure 2:**
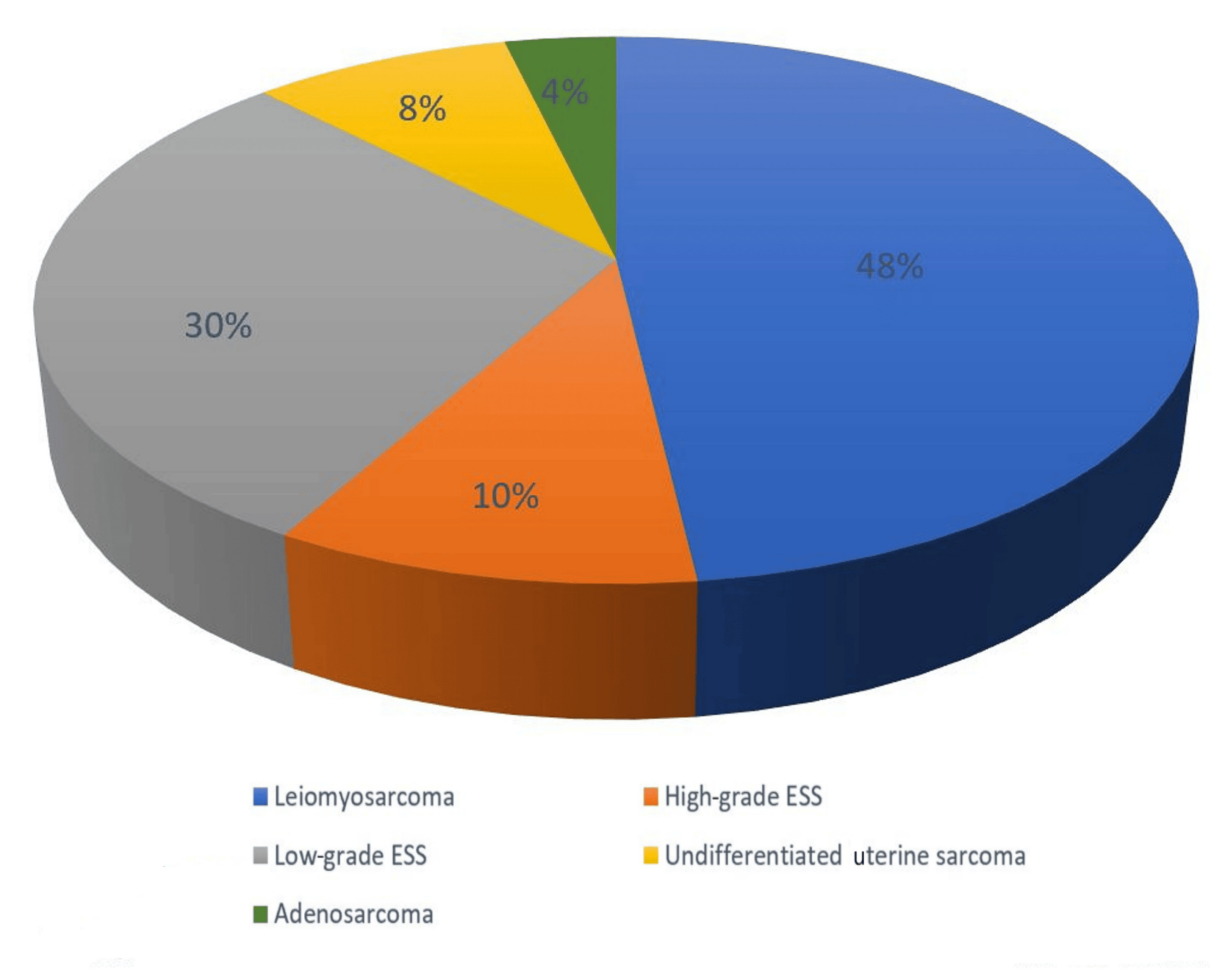
Distribution of cases according to histology ESS: endometrial stromal sarcoma

LMS (n=39; 48.1%) was the predominant histological subtype, followed by low-grade ESS (n=24; 29.6%), high-grade ESS (n=8; 9.9%), UUS (n=7; 8.6%), and adenosarcoma (n=3; 3.7%) (Figure 2).

Stage I disease was the most common (n=45; 55.6%), followed by stage II (n=10; 12.3%), stage III (n=12; 14.8%), and stage IV (n=14; 17.3%). UUS and high-grade ESS predominantly presented at advanced stages, while LMS was frequently diagnosed at stage I.

Primary surgery was performed in 74 patients (90%). Open surgery was performed in 69 patients (85.7%), while minimally invasive procedures, including robotic or laparoscopic approaches, were performed in 11 patients (14%). Hysterectomy with BSO was the most common procedure (n=64; 79%), with a minority undergoing myomectomy (n=3; 3.7%) or omentectomy (n=3; 3.7%).

A minority of patients (n=6; 7.4%) underwent pelvic or para-aortic lymphadenectomy, and one patient underwent large bowel resection with anastomosis. All patients referred from outside centers with a postoperative diagnosis of uterine sarcoma had residual disease, accounting for 19 patients (23.5%), arising from either incomplete pelvic tumor excision or a prior myomectomy. In several cases, distant metastases were present, likely reflecting inadequate preoperative metastatic workup.

Three cases of high-grade ESS (3.5%), three cases of LMS (3.5%), and one case of UUS (1.2%) presented at an advanced stage, accounting for seven patients (8.6%) of the total cohort. All these patients received neoadjuvant chemotherapy followed by interval debulking surgery.

Adjuvant therapy was administered to 40 patients (49.4%): chemotherapy in 26 (32.1%), radiotherapy in eight (9.9%), and hormonal therapy in six (7.4%). LMS was predominantly managed with chemotherapy, whereas low-grade ESS cases most commonly received hormonal therapy (Table 1).

**Table 1:** Clinicopathological characteristics of the cohort AUB: abnormal uterine bleeding; PMB: postmenopausal bleeding; LMS: leiomyosarcoma; LGESS: low-grade endometrial stromal sarcoma; HGESS: high-grade endometrial stromal sarcoma; Adeno: adenosarcoma; UUS: undifferentiated uterine sarcoma; NACT: neoadjuvant chemotherapy; BSO: bilateral salpingo-oophorectomy

Category		Frequency (n)	Percentage (%)
Demographics	Age (mean, years)	48	-
	BMI (mean, kg/m²)	24 (4.018)	-
Parity	Nulliparous	8	9.9
	Uniparous	9	11.1
	Multiparous	64	79
Menstrual status	Menstruating	26	32.1
	Perimenopausal	16	19.8
	Postmenopausal	39	48.1
Clinical presentation			
Symptoms	Abdominal pain	32	39.5
	PMB	12	14.8
	AUB	16	19.8
	Abdominal mass	21	25.9
Tumor characteristics			
Histology	LMS	39	48.1
	LGESS	24	29.6
	HGESS	8	9.9
	Adeno	3	3.7
	UUS	7	8.6
Stage	I	45	55.6
	II	10	12.3
	III	12	14.8
	IV	14	17.3
Residual disease	No	61	75.3
	Yes	20	23.7
Treatment			
NACT	No	74	90.4
	Yes	7	8.6
Mode of surgery	Open	70	85.7
	Laparoscopic	5	6.2
	Robotic	6	7.8
Morcellation	No	70	81.5
	Yes	11	13.6
Type of procedure	Myomectomy	3	3.7
	Hysterectomy only	5	6.2
	Hysterectomy+BSO	64	79
	Hysterectomy+BSO+omentectomy	3	3.7
	Other	6	7.4
Adjuvant treatment	No	41	50.6
	Chemotherapy	26	32.1
	Radiotherapy	8	9.9
	Hormonal therapy	6	7.4
Outcomes	Recurrence	36	44.4
Site of recurrence	Pelvis	17	-
	Lymph node	14	-
	Abdomen	11	-
	Peritoneum	8	-
	Lung	7	-
	Liver	4	-
Expired		43	53.1
Follow-up (median, months)		86	-
Median time of recurrence (median, months)		13	-
Overall survival (median, months)		72	-
Disease-free survival (median, months)		70	-

Recurrence 

Recurrence was observed in 36 patients (44.4%), with a median time to recurrence of 13 months. More than half of these patients experienced recurrence at multiple sites (n=20; 55%). The most frequent sites of recurrence were the pelvis (n=17), lymph nodes (n=14), abdomen (n=11), and peritoneum (n=8). Distant metastases involving the lung (n=7) and liver (n=4) were less common.

Residual disease after primary surgery (p=0.01), histologic subtype (p=0.02), and type of adjuvant therapy (p=0.001) were significantly associated with recurrence (Table 2).

**Table 2:** Correlation of recurrence with clinicopathological and treatment variable LMS: leiomyosarcoma; LGESS: low-grade endometrial stromal sarcoma; HGESS: high-grade endometrial stromal sarcoma; Adeno: adenosarcoma; UUS: undifferentiated uterine sarcoma; NACT: neoadjuvant chemotherapy; Chemo: chemotherapy

Variable	Recurrence (n=36)	No recurrence (n=45)	P-value
Mean age (years)	36±12.39	45±13.12	0.93
Mean BMI (kg/m²)	24.58±4.25	24.11±3.85	0.52
Morcellation			
Yes	3 (27.3%)	8 (72.7%)	0.19
No	33 (47.1%)	37 (52.9%)	
NACT			
Yes (7)	2 (28%)	5 (71%)	0.14
No (74)	34 (45.94%)	40 (54.06%)	
Residual disease after primary surgery			
Yes (19)	13 (68.4%)	6 (31.6%)	0.01
No (62)	23 (37.09%)	39 (62.3%)	
Stage			
Stage I	16 (35.6%)	29 (64.4%)	0.184
Stage II	6 (60%)	4 (40%)	
Stage III	8 (66.7%)	4 (33.3%)	
Stage IV	6 (42.9%)	8 (57.1%)	
Histology			
LMS	21 (53.8%)	18 (43.2%)	0.02
LGESS	4 (16.7%)	20 (83.3%)	
HGESS	4 (50%)	4 (50%)	
Adenosarcoma	2 (66.7%)	1 (33.3%)	
UUS	5 (71.4%)	2 (28.6%)	
Adjuvant treatment			
Observation	12 (29.3%)	29 (70.7%)	0.001
Chemo	21 (80.8%)	5 (19.2%)	
Radiotherapy	2 (25%)	6 (75%)	
Hormonal therapy	1 (16.7%)	5 (83.3%)	

Treatment of recurrence

Of the 36 patients with recurrence, 20 received chemotherapy, ranging from single-agent pazopanib to multi-agent regimens such as doxorubicin with ifosfamide or gemcitabine with docetaxel. Two patients underwent secondary debulking surgery. Four received combined radiotherapy and chemotherapy, while one received radiotherapy with hormonal therapy. Nine patients received palliative care.

Survival outcomes

**Figure 3:**
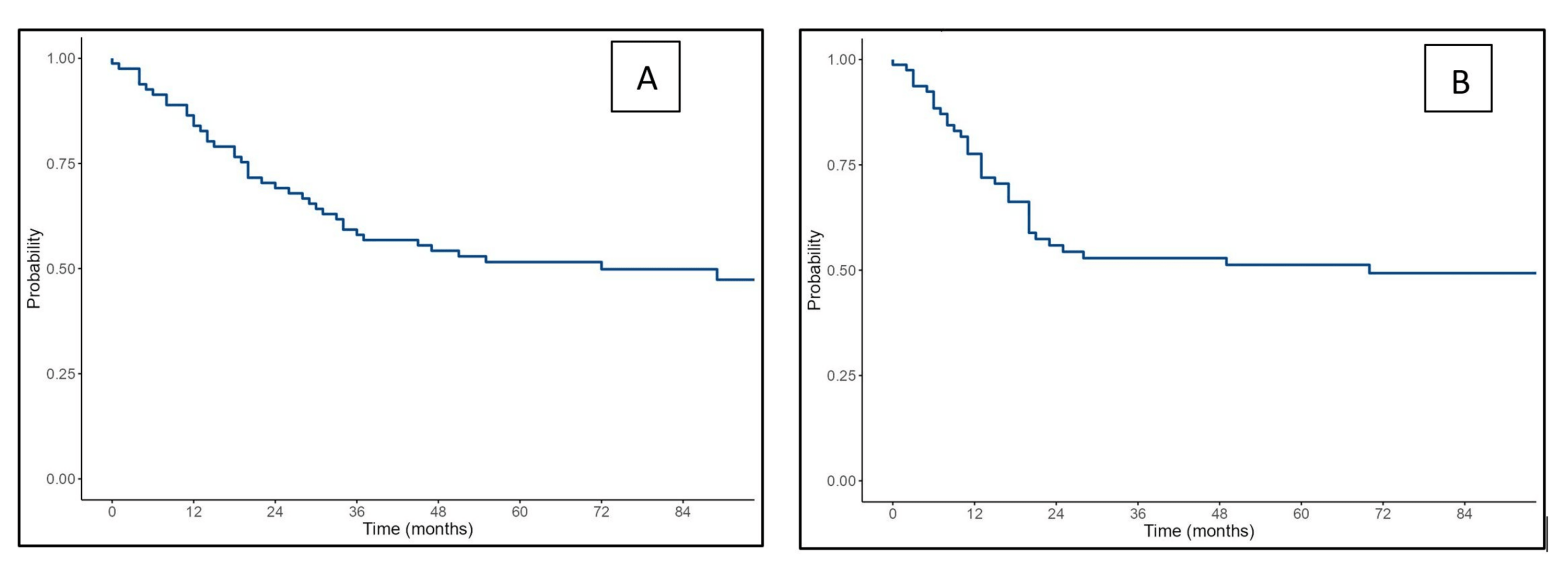
Kaplan-Meier survival curves of the study cohort showing (A) overall survival and (B) disease-free survival

The median follow-up duration was 86 months, during which 36 recurrences and 43 deaths occurred. The median OS was 72 months (95% CI: 34-not estimable), and the median DFS was 70 months (95% CI: 20-not estimable). Estimated OS rates at one, five, and seven years were 84% (95% CI: 76.3-92.3), 51.6% (95% CI: 41.7-63.8), and 49.8% (95% CI: 39.9-62.3), respectively. Corresponding DFS rates were 77.6% (95% CI: 68.8-87.6), 51.3% (95% CI: 40.8-64.4), and 49.3% (95% CI: 38.7-62.7), respectively (Figure 3).

**Figure 4:**
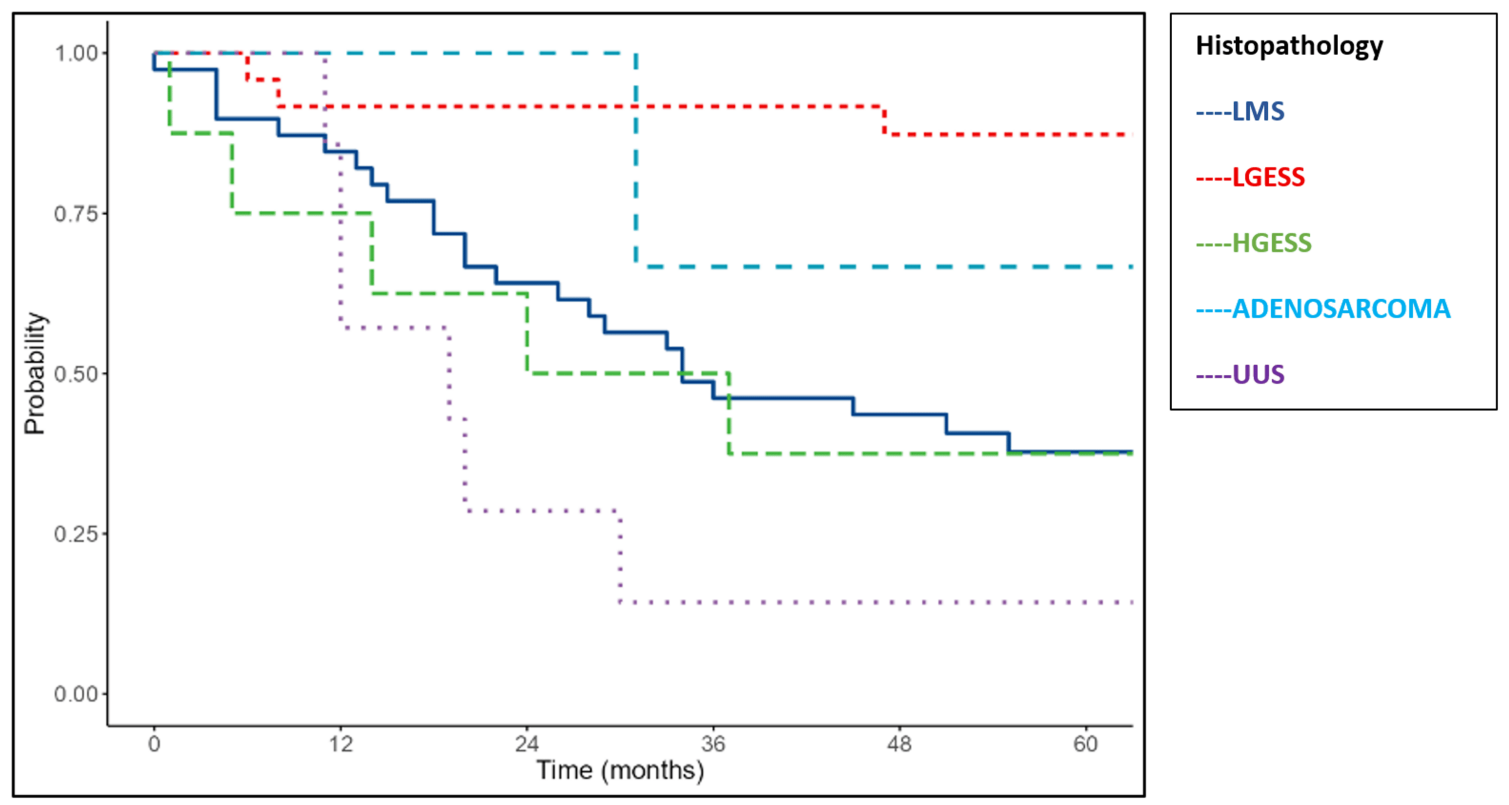
Kaplan-Meier survival curves of the study cohort showing overall survival according to histopathology LMS: leiomyosarcoma; LGESS: low-grade endometrial stromal sarcoma; HGESS: high-grade endometrial stromal sarcoma; UUS: undifferentiated uterine sarcoma

**Figure 5:**
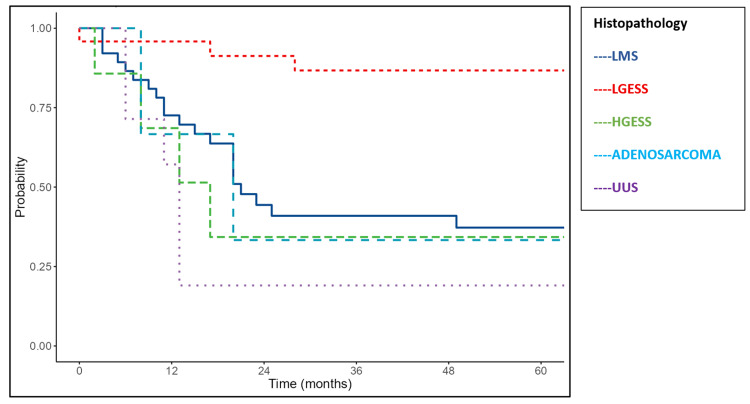
Kaplan-Meier survival curves of the study cohort showing disease-free survival according to histopathology LMS: leiomyosarcoma; LGESS: low-grade endometrial stromal sarcoma; HGESS: high-grade endometrial stromal sarcoma; UUS: undifferentiated uterine sarcoma

Histologic subtype was significantly associated with outcomes (p=0.004). LMS showed a median OS of 34 months and DFS of 21 months. High-grade ESS had poorer outcomes, with a median OS of 24 months and DFS of 17 months. Adenosarcoma demonstrated a median OS of 22 months and DFS of 20 months. UUS had the worst prognosis, with a median OS of 19 months and DFS of 13 months. In contrast, the median OS and DFS were not reached for low-grade ESS, reflecting favorable survival (Figures 4-5).

**Figure 6:**
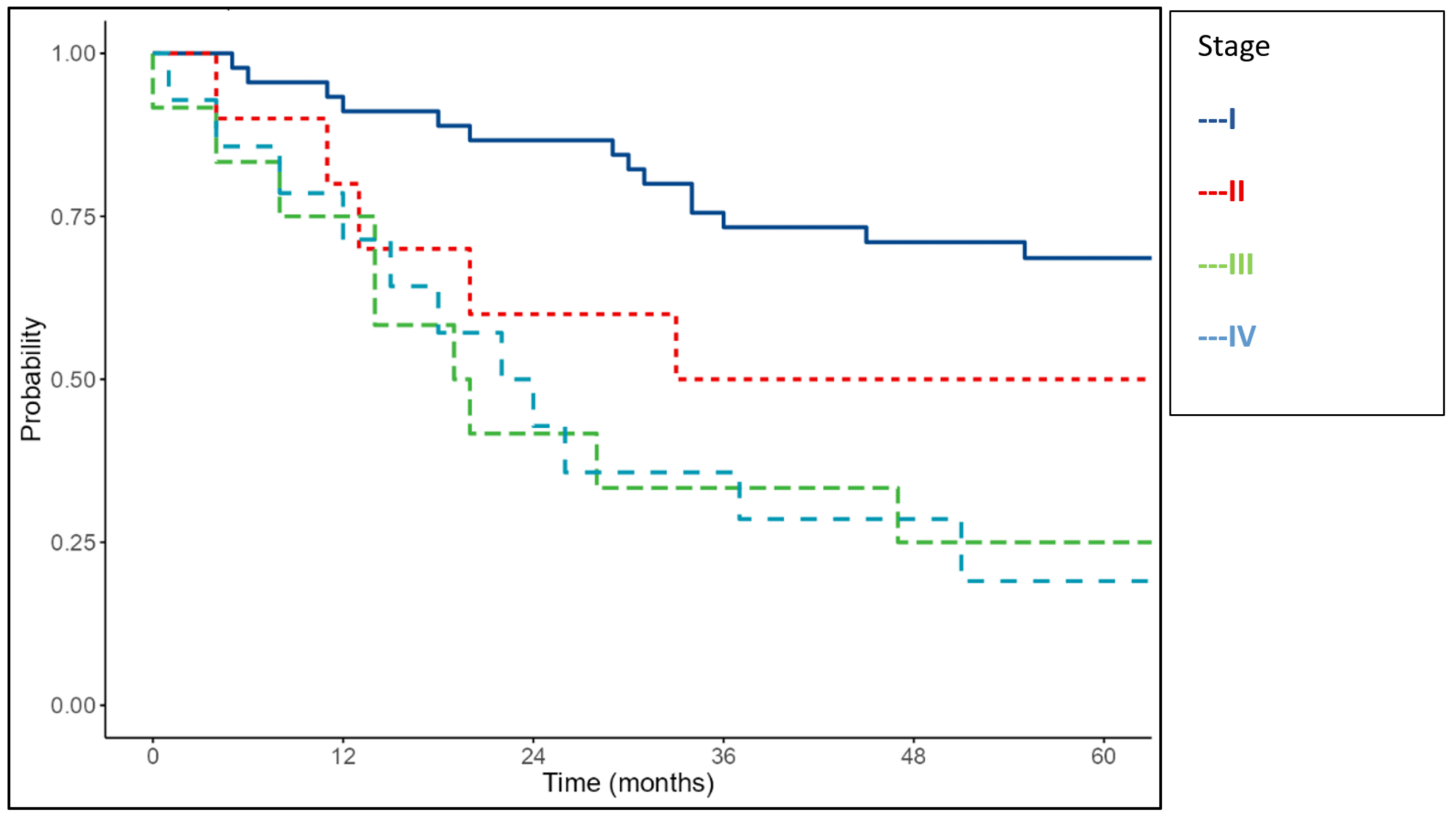
Kaplan-Meier survival curves of the study cohort showing overall survival according to FIGO stage FIGO: International Federation of Gynecology and Obstetrics

**Figure 7:**
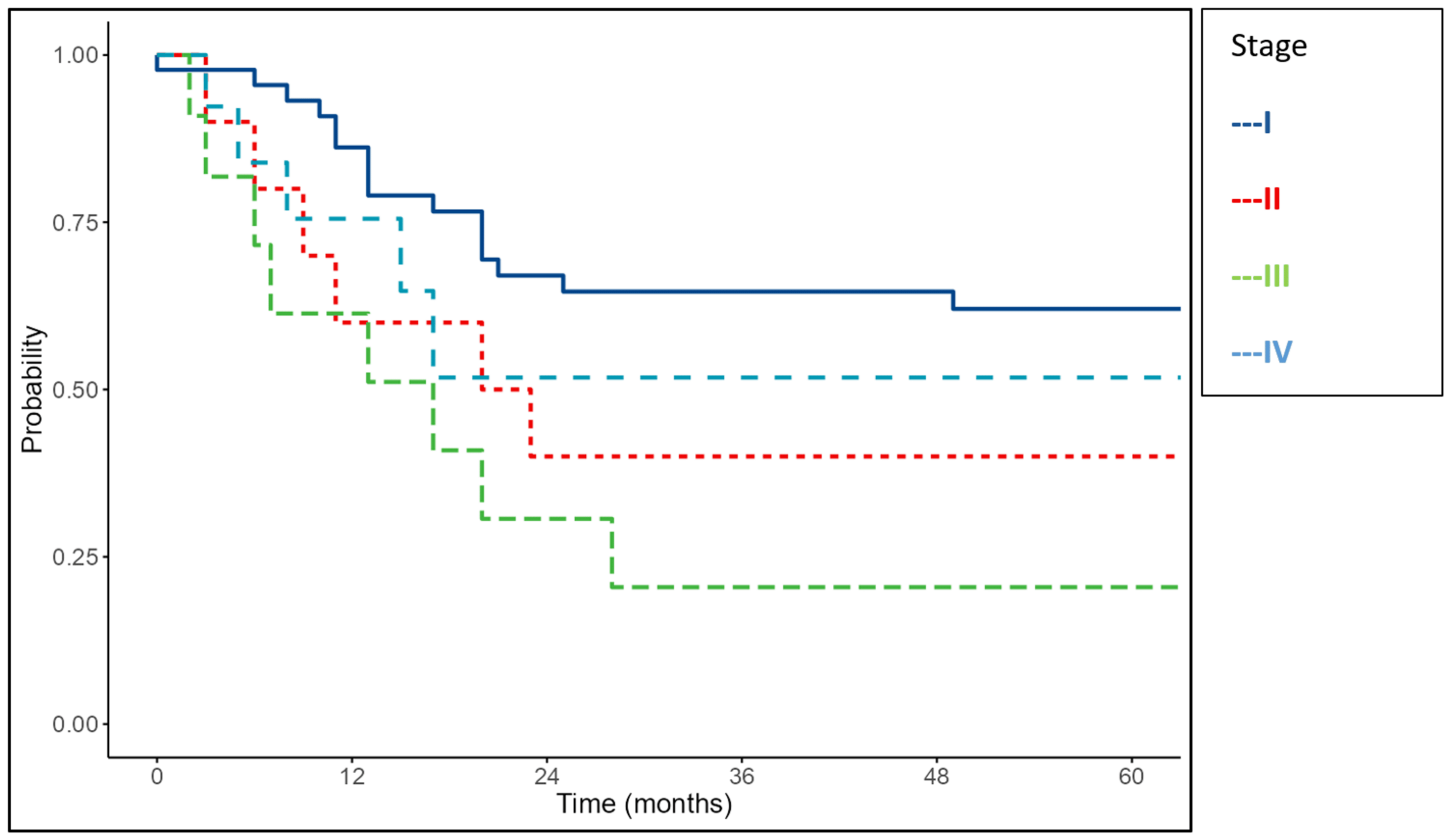
Kaplan-Meier survival curves of the study cohort showing disease-free survival according to FIGO stage FIGO: International Federation of Gynecology and Obstetrics

The FIGO stage also significantly affected survival. The median OS was 33 months for stage II, 19 months for stage III, and 22 months for stage IV, while the median OS for stage I was not reached (p<0.001). The median DFS was 20 months in stage II, 17 months in stage III, and 14 months in stage IV, whereas stage I DFS was not reached (p=0.025) (Figures 6-7).

**Figure 8:**
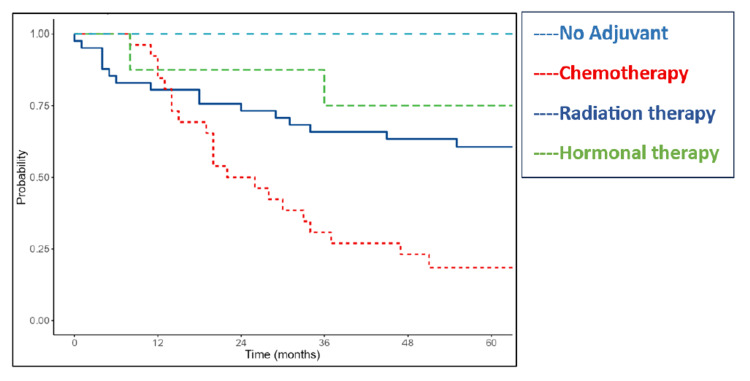
Kaplan-Meier survival curves of the study cohort showing overall survival according to adjuvant treatment

**Figure 9:**
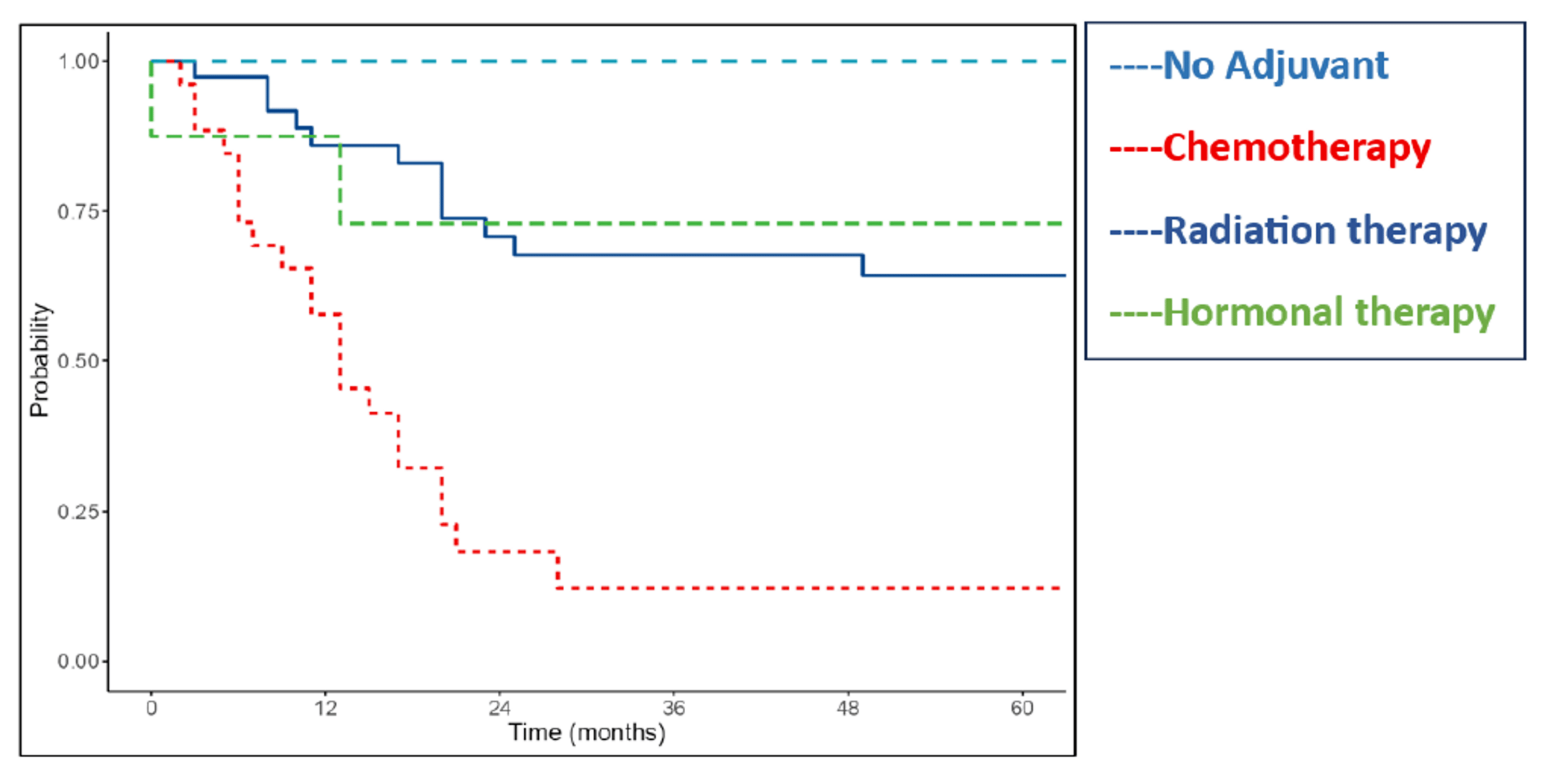
Kaplan-Meier survival curves of the study cohort showing disease-free survival according to adjuvant treatment

Adjuvant treatment further influenced outcomes (p=0.001). Patients receiving chemotherapy had a median OS of 22 months and DFS of 13 months. The median survival was not reached for patients in the radiotherapy and hormonal therapy groups due to limited events (Figures 8-9).

The survival outcomes of the cohort are shown in Table 3.

**Table 3:** Survival outcomes of the cohort OS: overall survival; DFS: disease-free survival; SE: standard error; LMS: leiomyosarcoma; LGESS: low-grade endometrial stromal sarcoma; HGESS: high-grade endometrial stromal sarcoma; Adeno: adenosarcoma; UUS: undifferentiated uterine sarcoma

Variable	OS		P-value	DFS		P-value
	Median (month)	SE		Median (month)	SE	
Stage						
I	Not reached	-	0.00	Not reached	-	0.025
II	33	41.1		20	9.48	
III	19	5.19		17	7.62	
IV	22	5.61		14	9.93	
Histology						
LMS	34	9.98	0.004	21	2.68	0.004
LGESS	Not reached	-		Not reached	-	
HGESS	24	16.26		17	5.12	
Adeno	22	9.12		20	9.79	
UUS	19	9.16		13	0.88	
Adjuvant treatment						
Chemotherapy	22	3.84	0.001	13	2.4	0.001
Radiation therapy	Not reached	-		Not reached	-	
Hormonal therapy	Not reached	-		Not reached	-	

Multivariable Cox regression analysis

Variables that were statistically significant on univariate analysis were entered into a multivariable Cox proportional hazards regression model using a backward conditional method to construct a parsimonious model.

For OS, tumor stage and type of adjuvant therapy remained independently associated with survival outcomes. Compared to observation, chemotherapy was not significantly associated with OS (HR: 0.94; 95% CI: 0.38-2.31; p=0.891). Radiation therapy was associated with a reduced hazard of death (HR: 0.22; 95% CI: 0.05-0.90; p=0.035), and hormonal therapy was also associated with lower mortality risk (HR: 0.11; 95% CI: 0.01-0.90; p=0.040). Advanced stage was independently associated with worse OS, with stage II (HR: 4.31; 95% CI: 1.45-12.76; p=0.008), stage III (HR: 5.42; 95% CI: 1.81-16.24; p=0.003), and stage IV (HR: 6.21; 95% CI: 2.32-16.66; p<0.001) demonstrating progressively increased mortality risk compared to stage I disease (Table 4). 

**Table 4:** Multivariable Cox regression analysis for overall survival

Variable	Adjusted hazard ratio (HR)	95% confidence interval	P-value
Adjuvant therapy (overall p=0.028)			
Observation	Reference		
Chemotherapy	0.94	0.38-2.31	0.891
Radiation therapy	0.22	0.05-0.90	0.035
Hormonal therapy	0.11	0.01-0.90	0.040
Tumor stage (overall p=0.002)			
Stage I	Reference		
Stage II	4.31	1.45-12.76	0.008
Stage III	5.42	1.81-16.24	0.003
Stage IV	6.21	2.32-16.66	<0.001

For DFS, type of adjuvant therapy remained independently associated with recurrence risk (overall p<0.001). Chemotherapy was associated with a higher hazard of recurrence (HR: 4.65; 95% CI: 2.24-9.68; p<0.001), likely reflecting treatment allocation in higher-risk disease. Radiation therapy and hormonal therapy were not significantly associated with DFS in the adjusted model (Table 5). 

**Table 5:** Multivariable Cox regression analysis for disease-free survival

Variable	Adjusted hazard ratio (HR)	95% confidence interval	P-value
Adjuvant therapy (overall p<0.001)			
Observation	Reference		
Chemotherapy	4.65	2.24-9.68	<0.001
Radiation therapy	0.82	0.18-3.66	0.794
Hormonal therapy	0.39	0.05-2.97	0.359

## Discussion

In this cohort, LMS was the most common subtype, with the majority of patients presenting at stage I and undergoing primary surgery, predominantly hysterectomy with BSO. Nearly half required adjuvant therapy, with chemotherapy favored for high-grade tumors and hormonal therapy for low-grade ESS. Recurrence occurred in 44% at a median of 13 months, and outcomes were significantly influenced by histology, FIGO stage, residual disease, and adjuvant therapy. LMS, high-grade ESS, and UUS demonstrated poor survival, whereas low-grade ESS had favorable outcomes. The median OS and DFS were not reached for stage I disease and low-grade ESS, while advanced stages and aggressive histologies had markedly worse prognoses.

The mean age at diagnosis in our cohort was 48±12 years, younger than that reported in Western populations (≈56 years) [12]. This likely reflects both regional demographic differences and the deliberate exclusion of carcinosarcoma, which usually presents in older women (mean 66.5 years) and can account for nearly half of uterine sarcoma cases [6]. The majority of our patients were postmenopausal, and the most common presenting symptoms were abdominal pain and an abdominal mass, consistent with previous studies describing vague and nonspecific presentations that often delay diagnosis [7].

Histologically, LMS (48.1%) was the most frequent subtype, followed by low-grade ESS, high-grade ESS, UUS, and adenosarcoma. This distribution is in line with prior reports, reinforcing LMS as the most prevalent uterine sarcoma worldwide [10]. At presentation, more than half of our patients had stage I disease, whereas UUS and high-grade ESS were usually advanced. Although stage is generally recognized as a key prognostic factor [13], we did not observe a significant association with recurrence. This discrepancy may be attributed to the modest sample size and the heterogeneity of histologic subtypes in our cohort. However, in multivariable analysis, tumor stage emerged as the strongest independent predictor of OS.

In line with existing literature, surgery was the primary treatment modality, with hysterectomy with BSO performed in 79% of cases. The role of lymphadenectomy in uterine sarcomas remains inconclusive, with current guidelines recommending the removal of only clinically or radiologically enlarged nodes [14]. In our cohort, lymphadenectomy was performed in 7% of patients. Management decisions in our cohort were guided by prevailing European Society of Gynaecological Oncology (ESGO) recommendations during the study period, although treatment was individualized through multidisciplinary tumor board evaluation based on stage, histology, and patient-specific factors.

Residual disease was identified in 23.5% of patients and was a strong predictor of recurrence, consistent with findings from the SARCUT study [15] and previous reports [16], both of which highlighted the critical role of complete resection. Notably, many patients with residual disease had undergone prior incomplete surgeries at outside centers, underscoring the adverse prognostic impact of suboptimal initial management.

Minimally invasive surgery was performed in 14% of cases, frequently involving morcellation. Although recurrence was not statistically associated with morcellation in our cohort, this aligns with observations from other studies [17], which reported inconclusive survival effects. Nevertheless, most studies suggest that morcellation worsens survival outcomes [9,18]. The risk of encountering an unsuspected LMS during surgery for presumed leiomyoma is estimated at one in 770 procedures. Current guidelines therefore advise the use of containment systems when morcellation cannot be avoided [19]. However, evidence indicates that even with containment, tumor dissemination occurs in nearly 9% of cases [20]. Taken together, these findings emphasize that improving survival depends on accurate preoperative diagnosis, careful evaluation of suspicious cases, and strict avoidance of morcellation whenever possible.

Adjuvant treatment was administered in half of our cohort. In multivariable analysis, radiotherapy was independently associated with improved OS, reflecting its role in locoregional control as reported in previous studies [21,22]. In contrast, chemotherapy did not demonstrate a significant association with OS in multivariable analysis, consistent with the limited efficacy observed in prior studies [23,24]. Hormonal therapy, administered exclusively in low-grade ESS, remained independently associated with improved OS in multivariable analysis, aligning with other studies [25] that support endocrine therapy in receptor-positive ESS.

Recurrence occurred in 44.4% of patients, with the pelvis and lymph nodes being the most common sites. The recurrence rate varied by histology, being highest in UUS (71.4%) and lowest in low-grade ESS (16.7%), closely paralleling findings from other studies [26], which demonstrated histology as a major determinant of recurrence risk.

Treatment of recurrence in our cohort was heterogeneous, with most patients receiving chemotherapy. However, some studies have highlighted the potential benefit of secondary cytoreduction at first recurrence, although further research is warranted to better define its role [27].

Survival outcomes in our series were comparable to international reports, with a median OS of 72 months and DFS of 70 months, falling within previously reported ranges (OS 50-80 months; DFS 40-70 months) [13,16]. Stage I disease and low-grade ESS were associated with favorable survival, while UUS, high-grade ESS, and adenosarcoma conferred poorer outcomes, consistent with other studies [26], which highlighted the prognostic significance of histologic subtype. Importantly, histology was significantly associated with survival on univariate analysis (p=0.004); however, it did not retain independent significance in the multivariable model.

Despite broad similarities with prior reports, several differences merit attention. Our patients were younger than those in the Western series, likely reflecting regional demographics and the exclusion of carcinosarcoma. In contrast to previously reported data [13], stage was not a significant prognostic factor in our cohort, possibly due to smaller case numbers. Radiotherapy appeared to offer benefit, but interpretation is limited by the small sample size. These variations highlight the importance of region-specific data and underscore the need for multi-institutional collaboration to refine management strategies for this rare malignancy.

The strengths of our study include a relatively large cohort with long follow-up, exclusion of carcinosarcoma allowing a focused evaluation of pure sarcomas, and a detailed analysis of recurrence patterns in relation to clinicopathological variables. Uniform slide review by a single expert oncopathologist further enhanced diagnostic consistency. Nonetheless, the retrospective design introduces inherent biases, including treatment selection bias. The heterogeneity of histological subtypes and therapeutic approaches, together with the rarity of uterine sarcomas, limits the statistical power of subgroup analyses.

Beyond these clinical observations, it is increasingly recognized that uterine sarcomas exhibit genomic alterations involving DNA repair, immune regulation, signalling pathways, and gene fusions, offering potential targets for therapy. Advances such as NTRK, PD-L1, and PARP inhibitors, along with molecular profiling and RNA sequencing, have refined tumor classification and enabled personalized treatment approaches [28]. While international guidelines exist, their implementation varies, particularly in resource-limited settings [20]. Continued progress relies on global collaboration and enrollment in biomarker-driven clinical trials [29].

By focusing exclusively on pure uterine sarcomas and excluding carcinosarcoma, this study provides a more accurate representation of their clinicopathological behavior and survival outcomes, offering a valuable benchmark for Indian data. In the absence of randomized trials, such real-world evidence is essential to guide treatment strategies, inform health policy, and complement emerging international guidelines. Ultimately, these findings underscore the need for prospective, multicenter collaborations and molecular characterization to refine risk stratification, standardize management, and improve therapeutic decision-making in this rare malignancy.

## Conclusions

Uterine sarcomas are rare, aggressive malignancies with heterogeneous clinicopathological features and survival outcomes. In this cohort, tumor stage and type of adjuvant therapy independently influenced OS on multivariable analysis, while histological subtype and residual disease were significant on univariate analysis. LMS, high-grade ESS, and UUS demonstrated poorer survival outcomes, whereas low-grade ESS showed a favorable prognosis. By excluding carcinosarcoma, this study provides a clearer representation of pure uterine sarcomas and offers region-specific data to inform evidence-based management. These findings underscore the importance of complete surgical resection, individualized adjuvant therapy, and the need for prospective, multicenter studies incorporating molecular characterization to refine treatment strategies and improve survival outcomes.
